# Child and Adolescent Mental Health Inpatient Referrals for Eating Disorders and/or Autism in the South of England, 2019–2025: Retrospective Cohort Study

**DOI:** 10.1192/bjo.2026.11229

**Published:** 2026-06-30

**Authors:** Gillian Combe, Samuel Lloyd-White, Daniel Cooper, Rohan Borschmann

**Affiliations:** 1 Oxford Health NHS Foundation Trust, Swindon, United Kingdom; 2 Oxford Health NHS Foundation Trust, Oxford, United Kingdom

## Abstract

**Aims:**

The prevalence of children and adolescents diagnosed with an eating disorder has recently increased, as has the prevalence of those diagnosed with autism. However, it is unknown whether there has been a corresponding increase in the proportion of young people admitted to inpatient mental healthcare facilities with eating disorders and/or autism in England. We aimed to: (1) examine referral patterns for child and adolescent mental health inpatient care involving a diagnosis of eating disorder and/or autism in the south of England from 2019–2025; and (2) describe the demographic and clinical characteristics of young people referred during this period.

**Methods:**

We conducted a prospective cohort study involving all young people (aged <18 years) referred for inpatient mental health treatment between 1 April 2019 and 31 March 2025 in one geographical area in the south of England. We collected data relating to demographic (age, sex, ethnicity) and clinical (diagnosis/es at admission and discharge, time between referral and admission, length of stay, and Mental Health Act status.

**Results:**

A total of 1,447 referrals were made (81.9% female, 83.3% White) and 1,022 (70.6%) were admitted. The proportion of young people admitted with an eating disorder diagnosis fluctuated between 35.8% and 54.0%, and the proportion admitted with an autism diagnosis ranged from 9.9% to 17.4%. The proportion admitted with comorbid autism and eating disorder diagnoses increased consistently year-on-year from 1.9% (2019–20) to 15.9% (2024–25). Between 2019 and 2021, the proportion of admissions involving: 1) a diagnosis of autism and/or eating disorder; 2) a nasogastric feeding tube; 3) use of the Mental Health Act; and 4) a referral categorised as urgent all increased markedly.

**Conclusion:**

We observed an eight-fold increase in the proportion of young people admitted with comorbid autism and eating disorder diagnoses between 2019 and 2025. We observed other trends in clinical presentations and outcomes, some of which may be related to the onset of the Covid-19 pandemic. Further research is needed to determine the longer-term trajectories and outcomes for young people referred for mental health inpatient care with a diagnosis of eating disorder and/or autism.

